# Balancing tuber vitamin C improvement with trade-off side effect by fine-tuning regulation of *StGGPs*

**DOI:** 10.1093/hr/uhag191

**Published:** 2026-05-13

**Authors:** Bo Zhang, Wei Luo, Saihang Zhang, Yang Zhong, Wenjuan Xie, Di Hu, Xiaofeng Xue, Jinzhe Zhang, Yi Shang, Jianfei Xu, Ling Ma

**Affiliations:** Engineering Research Center for Valorization of Unique Bio-Resources in Yunnan, Ministry of Education, Yunnan Normal University, Kunming, China; Shenzhen Branch, Guangdong Laboratory of Lingnan Modern Agriculture, Genome Analysis Laboratory of the Ministry of Agriculture and Rural Affairs, Agricultural Genomics Institute at Shenzhen, Chinese Academy of Agricultural Sciences, Shenzhen, China; Institute of Vegetables and Flowers, Chinese Academy of Agricultural Sciences/Key Laboratory of Biology and Genetic Improvement of Tuber and Root Crops/Key Laboratory of Biology and Genetic Improvement of Horticultural Crops, Ministry of Agriculture and Rural Affairs, Beijing, China; College of Life Science and Technology, Huazhong Agricultural University, Wuhan, China; Southwest United Graduate School, Kunming, China; Engineering Research Center for Valorization of Unique Bio-Resources in Yunnan, Ministry of Education, Yunnan Normal University, Kunming, China; Shenzhen Branch, Guangdong Laboratory of Lingnan Modern Agriculture, Genome Analysis Laboratory of the Ministry of Agriculture and Rural Affairs, Agricultural Genomics Institute at Shenzhen, Chinese Academy of Agricultural Sciences, Shenzhen, China; Shenzhen Branch, Guangdong Laboratory of Lingnan Modern Agriculture, Genome Analysis Laboratory of the Ministry of Agriculture and Rural Affairs, Agricultural Genomics Institute at Shenzhen, Chinese Academy of Agricultural Sciences, Shenzhen, China; Dehong Agricultural Technology Extension Center, Dehong Agricultural Science Institute, Yunnan, China; Engineering Research Center for Valorization of Unique Bio-Resources in Yunnan, Ministry of Education, Yunnan Normal University, Kunming, China; Institute of Apicultural Research, Chinese Academy of Agricultural Sciences, Beijing, China; Institute of Vegetables and Flowers, Chinese Academy of Agricultural Sciences/Key Laboratory of Biology and Genetic Improvement of Tuber and Root Crops/Key Laboratory of Biology and Genetic Improvement of Horticultural Crops, Ministry of Agriculture and Rural Affairs, Beijing, China; Engineering Research Center for Valorization of Unique Bio-Resources in Yunnan, Ministry of Education, Yunnan Normal University, Kunming, China; Institute of Vegetables and Flowers, Chinese Academy of Agricultural Sciences/Key Laboratory of Biology and Genetic Improvement of Tuber and Root Crops/Key Laboratory of Biology and Genetic Improvement of Horticultural Crops, Ministry of Agriculture and Rural Affairs, Beijing, China; Engineering Research Center for Valorization of Unique Bio-Resources in Yunnan, Ministry of Education, Yunnan Normal University, Kunming, China

## Abstract

Potato (*Solanum tuberosum* L.) is a globally important staple crop. Improving its nutritional quality without compromising yield—a classic manifestation of the growth–defense trade-off—remains a major challenge in modern agriculture. Traditional breeding in potato is hindered by its highly heterozygous genome and tetrasomic inheritance, making it difficult and time-consuming to introduce and fix new traits. To address this, we applied a gene-editing approach to enhance vitamin C (Vc) content in tubers by mutating the upstream open reading frames of two potato *GDP-l-galactose phosphorylase* genes. New variants, with significantly elevated Vc levels, well-balanced nutrient profiles, and maintained yield, were created. The nutritionally enriched​ tubers retained high Vc content after culinary processing and exhibited substantially increased antioxidant activity. Importantly, we also found that excessive Vc enrichment can disrupt auxin activity, leading to developmental arrest and yield penalties. Thus, our study not only provides an effective strategy for multitrait improvement in potato, but also offers broader insights into the physiological mechanisms governing the balance between yield and nutritional quality in horticultural crops.

## Introduction

Improving the nutritional quality of horticultural crops without compromising yield—a classic manifestation of the growth–defense trade-off—remains a major challenge in modern agriculture [1, 2]. As a staple crop central to global food security, potato serves as a key dietary source of both energy and essential nutrients [3, 4]. Notably, the potato tuber is a valuable natural source of vitamin C (Vc), an essential antioxidant nutrient that plays a crucial role in regulating cellular redox potentials [5]. Because humans cannot synthesize Vc endogenously, dietary intake is paramount for health [6, 7], making the enhancement of Vc content in crops a critical strategy for improving nutritional security [4, 8]. In plants, Vc, also known as ascorbate, fulfills multifaceted physiological roles: beyond serving as the primary antioxidant for scavenging reactive oxygen species (ROS) and maintaining redox homeostasis [9], it acts as a cofactor for enzymes involved in hormone biosynthesis and cell wall metabolism [10], and participates in the regulation of developmental processes such as cell division and elongation [11]. This dual significance—for both human nutrition and plant health—underpins the rationale for enhancing Vc content in crops.

Conventional breeding for high Vc in potato is impeded by its highly heterozygous, tetraploid genome [12]. While new strategies like hybrid diploid breeding and gene editing are emerging [13, 14], successful enrichment must reconcile metabolic enhancement with agronomic performance [15, 16]. Conventional metabolic engineering, frequently relying on strong constitutive overexpression, often disrupts cellular homeostasis and incurs yield penalties [17, 18]. As an alternative, fine-tuning endogenous gene expression offers a more precise strategy. GDP-l-galactose phosphorylase (GGP) catalyzes the rate-limiting step of the primary Vc biosynthesis pathway [5, 19] and is post-transcriptionally regulated by a conserved upstream open reading frame (uORF) in its 5′ leader [20]. Precise editing of this uORF presents a cis-regulatory strategy to enhance translation of the native *GGP* transcript. While this approach has proven effective in leafy vegetables [21, 22], its application in tuber crops—and its potential to bypass the yield–quality trade-off—has remained unexplored.

The pivotal phytohormone auxin governs diverse aspects of plant growth and development, including organ patterning, cell division, and response to the environment, making it a central regulator of crop architecture and yield [23]. Its biosynthesis, transport, and signaling pathways have been extensively studied in model systems, and emerging research in crops like rice, maize, and tomato highlights how targeted manipulation of auxin-related genes can directly improve traits such as grain size [24, 25], root architecture [26], and fruit development [27]. Here, we test the hypothesis that CRISPR-mediated uORF editing of *StGGP* genes can increase tuber Vc content without growth impairment. By generating a series of potato lines with varying degrees of Vc enrichment, we assessed outcomes across an accumulation gradient. We demonstrate that moderate uORF editing yields nutritionally enhanced potatoes with no detectable trade-offs, whereas extreme Vc elevation reveals a metabolic ceiling beyond which growth is compromised.

Importantly, our findings uncover a feedback mechanism between Vc and auxin that underlies this threshold. While auxin is known to suppress Vc biosynthesis [28, 29], we show that Vc hyperaccumulation disrupts auxin homeostasis, establishing a bidirectional inhibitory circuit. This mechanism delineates a physiological boundary for engineering metabolic traits without agronomic cost. By identifying this equilibrium, our study offers a generalizable framework for crop trait enhancement, transitioning from proof-of-concept toward a strategic principle for sustainable enrichment.

## Results

### Engineering a tunable vitamin C enrichment spectrum in potato by uORF editing

**Figure 1:**
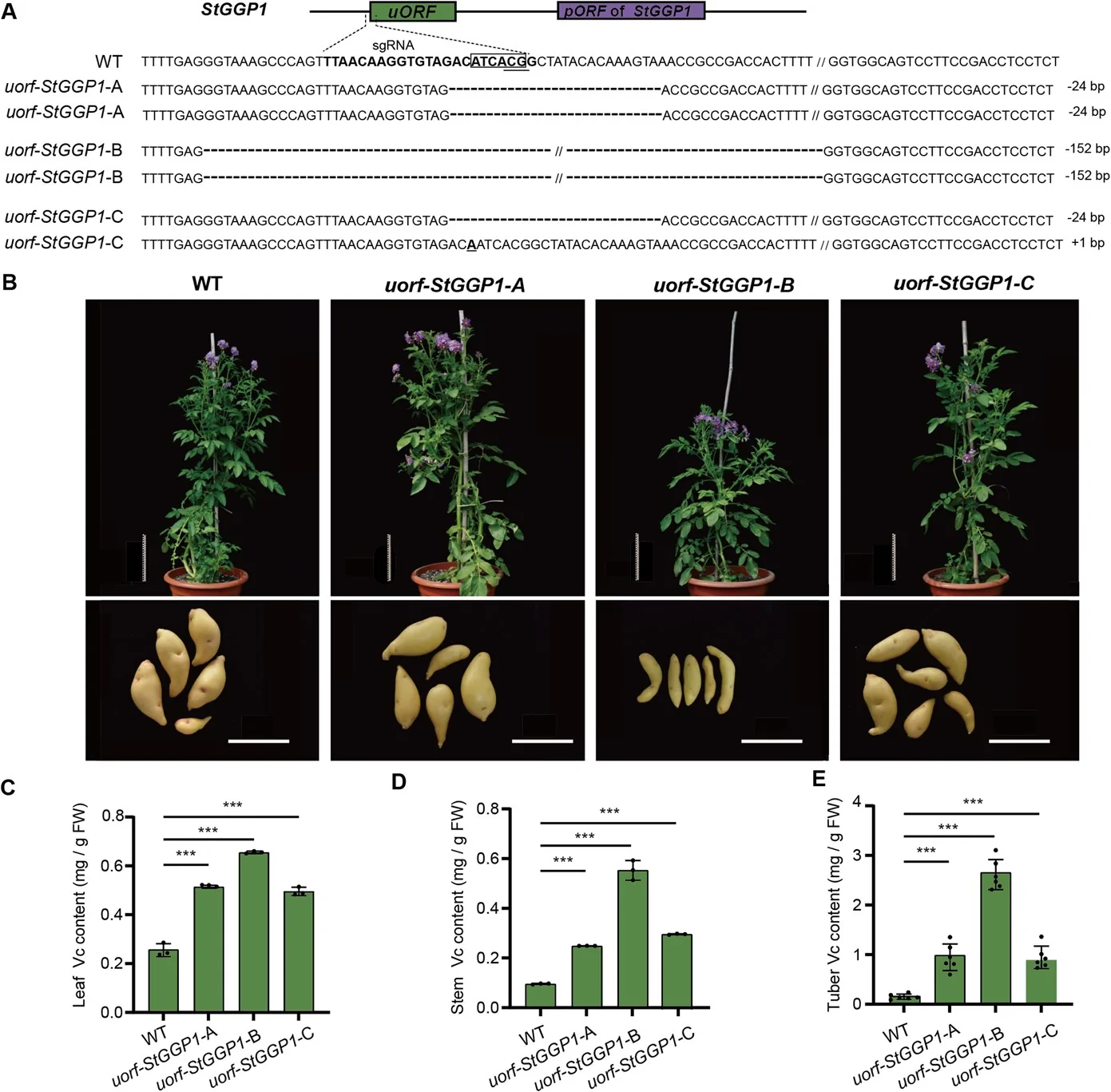
CRISPR/Cas9-mediated editing of *uorf-StGGP1*. (A) Sequence alignments of WT and the *uorf-StGGP1* edited lines. The *uorf-StGGP1* region and downstream primary ORF (pORF-*StGGP1*) are indicated by boxes. The WT sequence is shown at the top, with the sgRNA target (bold) and protospacer-adjacent motif (PAM, underlined). The *uorf-StGGP1* start codon is marked by a rectangle. Edited nucleotides are numbered (deletions: hyphens; insertions: bold and underlined). (B) Growth status of WT and *uorf-StGGP1* edited lines, and harvested tubers. Scale bar = 20 cm. (C–E) Comparison of Vc content in (C) leaf, (D) stem, (E) tuber flesh of WT and edited lines. Values = means ± SD (leaf/stem: *n* = 3; tuber: *n* = 6). Data were analyzed by one-way ANOVA followed by Dunnett’s *post hoc* test. ^***^P <  0.001 vs WT control; ns, not significant.

To systematically investigate the trade-off between Vc enrichment and agronomic performance in potato, we employed a precision genome editing strategy targeting the uORFs of two key Vc biosynthetic genes—*StGGP1* and *StGGP2*. These uORFs are highly conserved within the Solanaceae family (Fig. 1A; Fig. S1).

In the diploid potato S15–65, editing of *StGGP1* generated three independent mutants, including two homozygous lines (*uorf-StGGP1*-A, with a 24-bp deletion; *uorf-StGGP1*-B, with a 152-bp deletion) and one heterozygous line (*uorf-StGGP1*-C, with a 24-bp deletion and a 1-bp insertion) (Fig. 1A and B; Fig. S2A–D). Tuber Vc levels increased by 8.72-, 24.12-, and 7.35-fold in *uorf-StGGP1*-A, B, and C, respectively compared to the wild type, with significant rises also detected in leaves and stems (Fig. 1C–E). The 24.12-fold Vc increase in tubers of *uorf-StGGP1*-B thus represents, to our knowledge, the highest accumulation reported to date, surpassing the previously recorded Vc enhancement—a 6.2-fold increase—in tomato by overexpressing a kiwifruit *GGP* gene [17] (Table S1). Notably, this line displayed visible growth impairment, including reduced plant height and tuber weight (Fig. 1B), which suggests a potential trade-off between extreme Vc enrichment and normal growth. Our results establish uORF editing as a potent tool for creating a tunable spectrum of Vc accumulation in potato.

**Figure 2:**
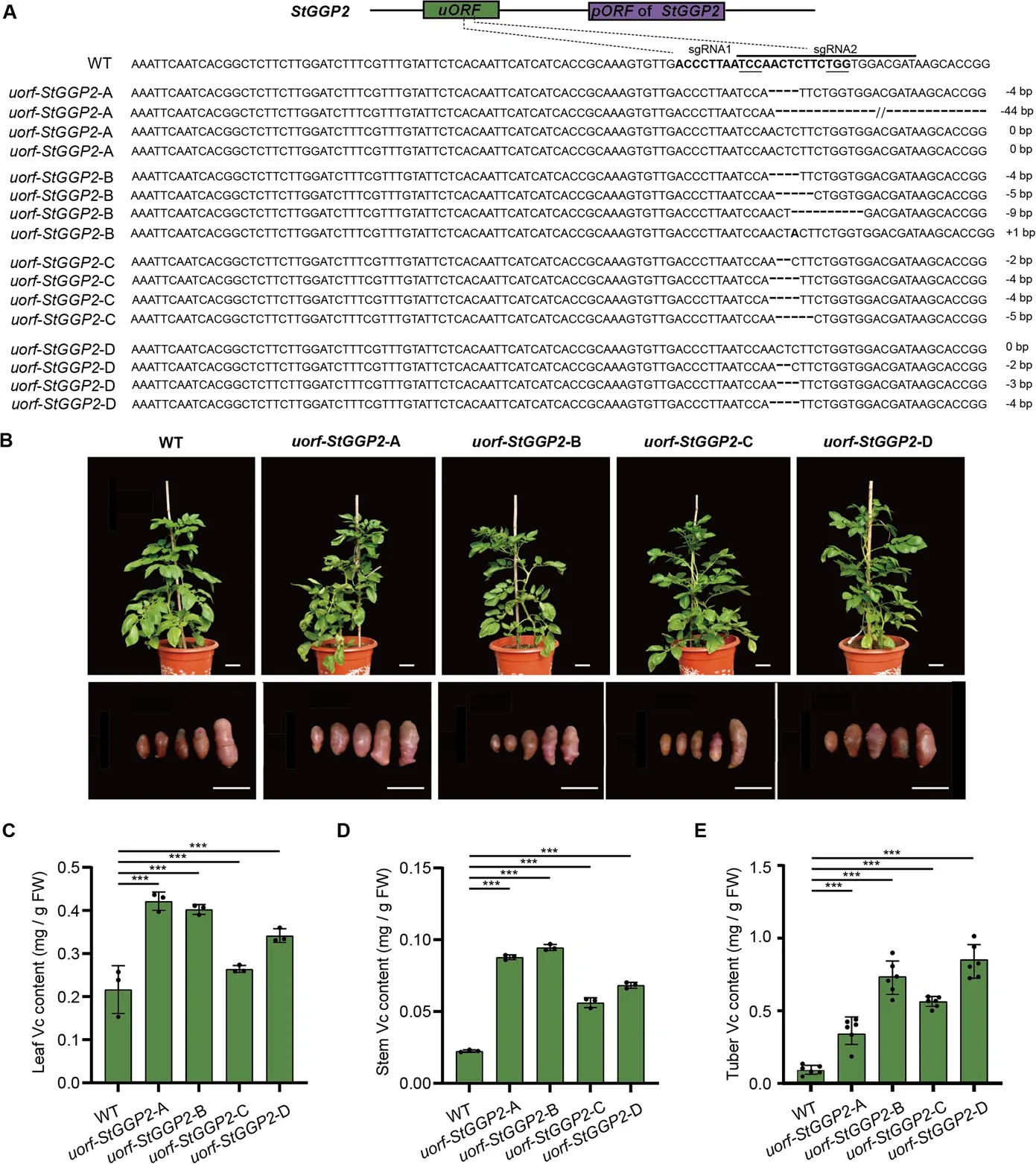
CRISPR/Cas9-mediated editing of *uorf-StGGP2.*  **(**A) Sequence alignments of WT and *uorf-StGGP2* edited lines. The *uorf-StGGP2* region and downstream pORF-*StGGP2* are indicated by boxes. The WT sequence (top) shows sgRNA1(bold) and sgRNA2 (underlined) targets, and the protospacer-adjacent motif (underlined). The *uorf-StGGP2* start codon is marked by a rectangle. Edited nucleotides are numbered (deletions: hyphens; insertions: bold). (B) Growth phenotype of WT and edited lines, and harvested tubers. Scale bar = 10 cm. (C–E) Comparison of Vc content in (C) leaf, (D) stem, (E) tuber flesh of WT and edited lines. Values = means ± SD (leaf/stem: *n* = 3; tuber: *n* = 6). Data were analyzed by one-way ANOVA followed by Dunnett’s *post hoc* test. ^***^P < .001 vs WT control; ns, not significant.

For *StGGP2*, initial editing in S15–65 produced four mutant lines, but these were unexpectedly tetraploid—likely due to genome instability during regeneration [30]—and were excluded from further analysis. We then designed sgRNAs targeting regions distal to the uORF start codon and successfully generated four chimeric edited lines (*uorf-StGGP2*-A to -D) in the tetraploid cultivar Désirée (Fig. 2A and B, Fig. S2E–I). These lines carried various deletions (2–44 bp), with *uorf-StGGP2*-B also containing a 1-bp insertion (Fig. 2A). Elevated Vc levels were also consistently observed in the leaves and stems of all four edited lines (Fig. 2C and D). Notably, the tuber Vc content in these mutants was significantly enhanced, showing 3.79- to 9.47-fold increases over the wild-type *Désirée* (Fig. 2E).

Collectively, we have established a comprehensive set of plant materials exhibiting a broad gradient of Vc accumulation, providing an ideal experimental system for dissecting the physiological and molecular consequences of metabolic engineering in a staple crop.

### A dose-dependent effect of uORF edits on vitamin C accumulation

**Figure 3:**
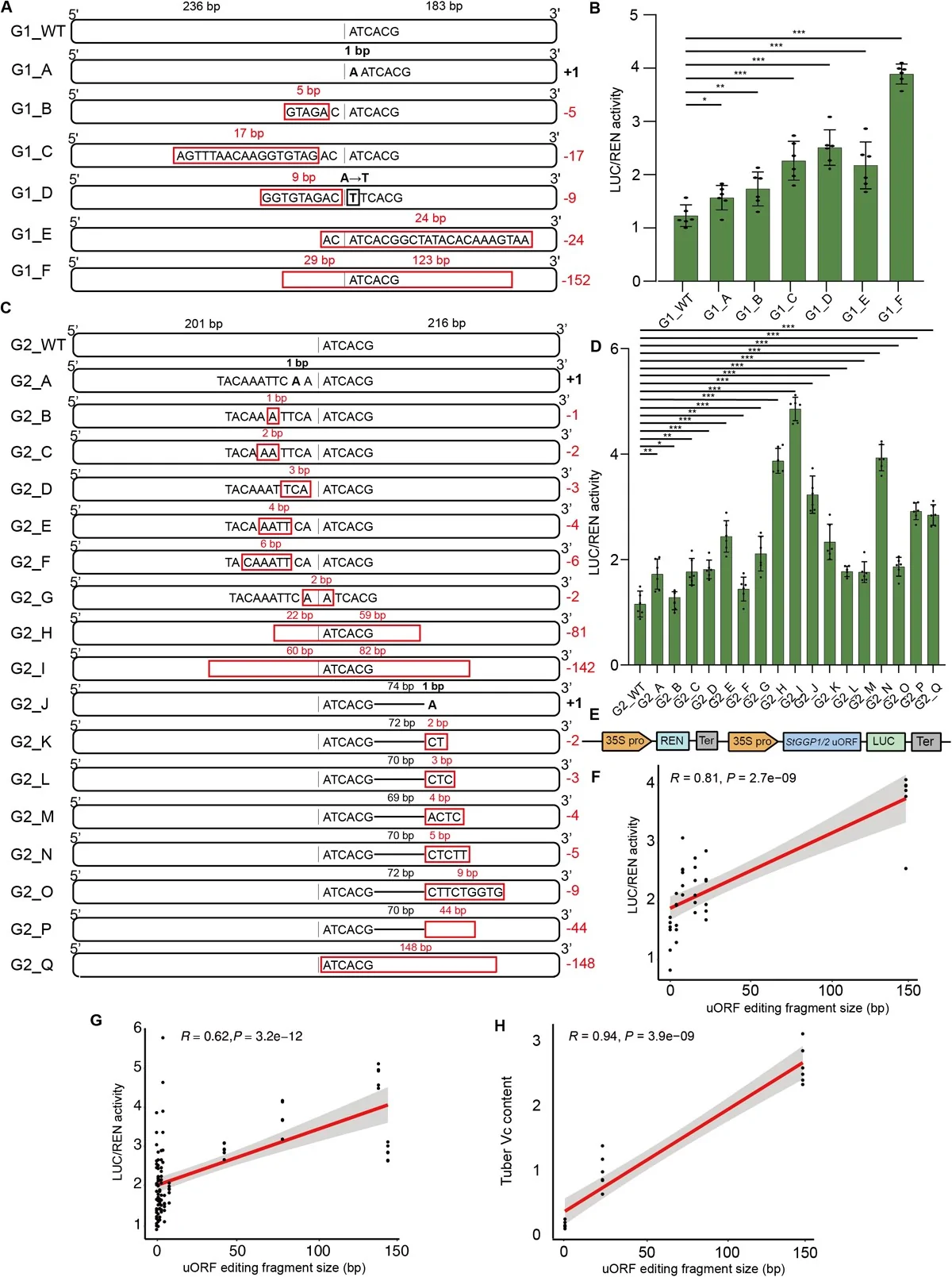
Effects of *uorf-StGGPs* mutations on LUC/REN activity. **(**A) Six *uorf-StGGP1* mutations (A–F) tested via dual-luciferase assay. ATCACG denotes the putative *uorf-StGGP1* initiation codon. Deleted/inserted nucleotides are numbered and marked by rectangles and bold font, respectively; a start codon mutation is indicated by a rectangle and bold font. (B) Effects of the six *uorf-StGGP1* mutations on LUC/REN activity. (C) Seventeen *uorf-StGGP2* mutations (A–Q) tested via dual-luciferase assay. ATCACG denotes the putative *uorf-StGGP2* initiation codon. Deleted/inserted nucleotides are numbered and marked by rectangles and bold font, respectively. (D) Effects of the seventeen *uorf-StGGP2* mutations on LUC/REN activity. (E) Schematic of the dual-luciferase assay construct. (F–G) Positive correlation between the uORF editing fragment size (bp; representing the net sequence change of deletion or insertion) and the normalized LUC/REN activity ratio of *uorf-StGGP1* (F) and *uorf-StGGP2* (G). (H) Positive correlation between tuber Vc content (data from Fig. 1C) and the uORF editing fragment size (data from Fig. 1A) in the *uorf-StGGP1* homozygous edited lines. Data were analyzed by one-way ANOVA followed by Dunnett’s *post hoc* test. ^*^P <  0.05, ^**^P <  0.01, ^***^P <  0.001 vs WT control; ns, not significant.

We next asked whether the extent of Vc enhancement could be fine-tuned by the size of the uORF deletion. Although knocking out the start codon of *uORF-GGPs* has been shown to elevate Vc [21, 22], it remained unknown whether a dose-dependent relationship exists. Using a dual-luciferase reporter assay [20], we found that larger deletions within the uORFs of both *StGGP1* and *StGGP2* progressively increased the translational efficiency (Fig. 3A–D), establishing a clear molecular dose–response at the translational level.

To move beyond the correlation between deletion size and phenotype and establish a true, quantitative dose–response relationship, we engineered a panel of precise point mutants designed to systematically weaken the translation initiation efficiency of the *StGGP1* (G1) and *StGGP2* (G2) uORFs. This series targeted the key regulatory elements—the Kozak sequence context and the start codon—to create a gradient of uORF ‘strength’ (Fig. S3). For *StGGP1*, weakening the +4 Kozak context (G1-M2) produced the strongest reduction in uORF-mediated translational repression. In contrast, for *StGGP2*, the combined mutation of the −3 Kozak context and the start codon (G2-M3) was most potent. Both datasets demonstrated a graded, stepwise increase in downstream reporter translation (LUC/REN activity) as uORF strength decreased, with the introduction of an early stop codon (complete uORF ablation) establishing the ‘zero uORF’ functional baseline (Fig. S3). These results reveal that uORF-mediated translational repression is finely tunable via defined molecular interventions, confirming a causal dose–response mechanism underlying the phenotypic observations.

This molecular gradient was directly reflected in planta for *StGGP1*. In homozygous edited lines, a strong positive correlation was observed between the uORF editing fragment size and tuber Vc content: a 152-bp deletion (*uorf-StGGP1*-B) led to a 24.12-fold increase, significantly surpassing the effect of a 24-bp deletion (*uorf-StGGP1*-A, 8.72-fold) (Fig. 3E, F, and  H). For *StGGP2*, a definitive correlation in tubers could not be established due to the chimeric nature of the tetraploid edited lines, despite the positive trend in the reporter assay (Fig. 3G).

At the molecular level, uORF editing also enhanced both transcript and protein abundance. The variant with the largest deletion (*uorf-StGGP1*-B) showed the most pronounced increase in mRNA levels (Fig. S4A), suggesting potential effects on mRNA stability or feedback regulation. The majority of the tested mutants showed an increase in GGP protein levels (Fig. S4B and C). These findings indicate that uORF editing boosts Vc biosynthesis by coordinately increasing translational efficiency, mRNA accumulation, and ultimately protein abundance.

This precise dose–response relationship demonstrates that uORF editing provides a tunable mechanism to predictably modulate metabolic flux. Having established this spectrum of Vc accumulation, we next assessed its potential agronomic trade-offs.

### Defining an agronomic trade-off-free window for vitamin C enrichment

**Figure 4:**
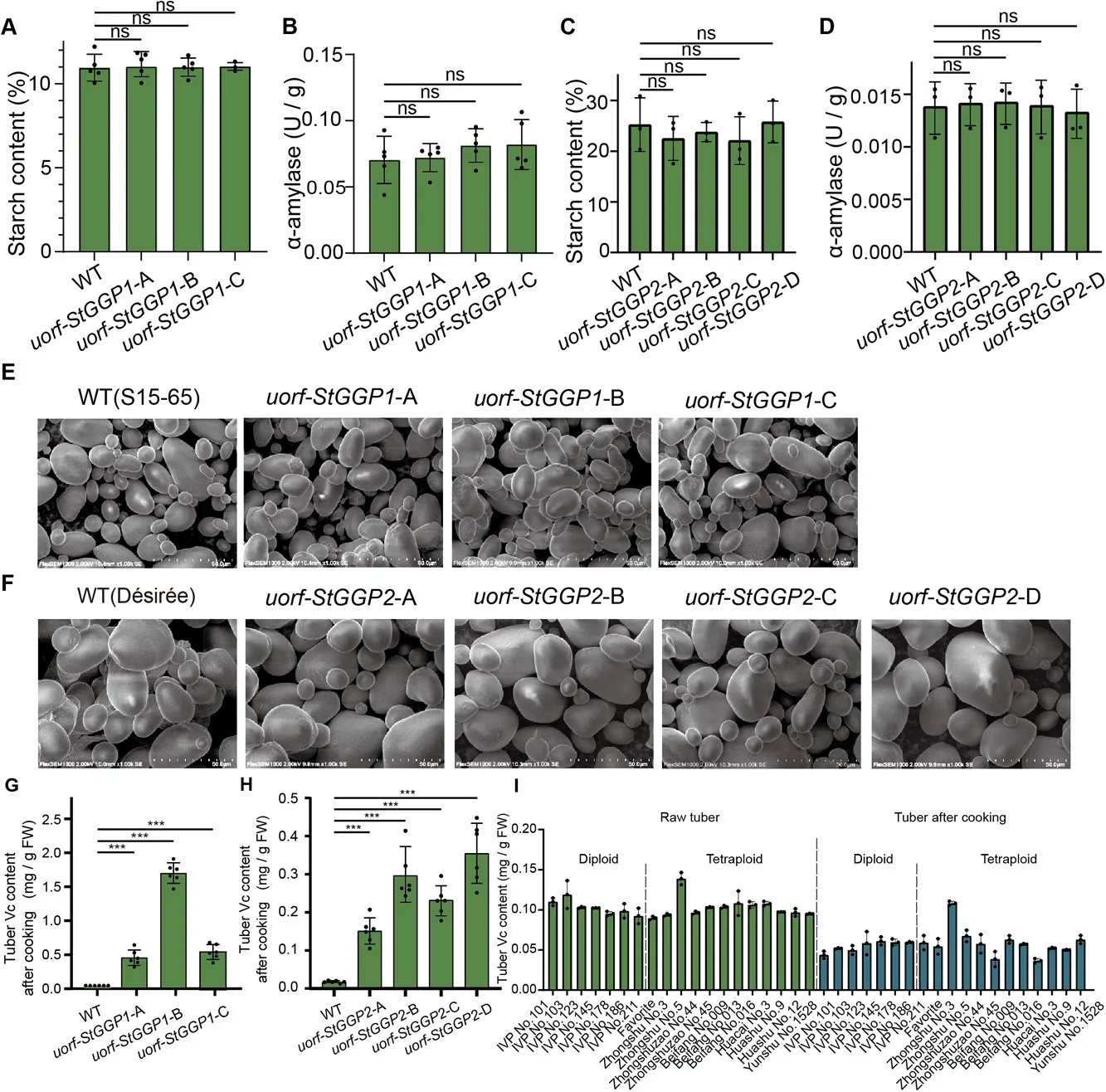
Starch content and α-amylase activity in *uorf-StGGP1/2*-edited lines. (A, B) Starch content (A) and α-amylase activity (B) in *uorf-StGGP1*-edited vs WT. All values: mean ± SD (*n* = 5 biological replicates). (C and D) Starch content (C) and α-amylase activity (D) in *uorf-StGGP2*-edited vs WT. All values: mean ± SD (*n* = 3 biological replicates). (E and F) Microscopic images of *uorf-StGGP1* (E)- and *uorf-StGGP2* (F)-edited vs WT plants. (G and H) Vc content in cooked potato flesh of WT vs *uorf-StGGP1*(H)/*-2* (G) edited lines. (I) Vc content in raw and processed tubers of diploid/tetraploid lines. Data were analyzed by one-way ANOVA followed by Dunnett’s *post hoc* test. ^*^P <  0.05, ^**^P <  0.01, ^***^P <  0.001 vs WT control; ns, not significant.

To critically assess potential trade-offs between nutritional enhancement and agronomic performance, we conducted a rigorous assessment of key traits in the gene-edited potato lines. Strikingly, moderate Vc enrichment—as achieved in the *uorf-StGGP1*-A line (8.72-fold increase) and *Désirée*-based *StGGP2* lines (up to 9.47-fold increase)—did not affect tuber yield, starch content, or α-amylase activity, relative to wild-type controls (Fig. 4A–D; Fig. S5). Scanning electron microscopy confirmed that starch granule morphology was preserved in all edited lines, with granules retaining their characteristic polyhedral shape and smooth surfaces (Fig. 4E and F). Granule size distribution and surface integrity were similarly unaffected, indicating that elevated Vc biosynthesis does not disrupt starch physical structure—a key determinant of texture and functionality in processed potato products [31, 32].

Given the susceptibility of Vc to thermal degradation [33, 34], we assessed its retention after cooking to determine nutritional relevance. Although steaming for 30 min reduced Vc levels across all genotypes, edited lines maintained significantly higher concentrations (0.19–1.72 mg/g FW) than the WT (Fig. 4G and H). This thermal stability was consistent across genetic backgrounds (Fig. 4I), suggesting that higher initial Vc concentrations may enhance heat resistance. Notably, a 100-g serving of steamed enriched tubers provided approximately 95 mg of Vc—comparable to a raw kiwifruit and sufficient to meet the recommended daily intake of 90 mg [6, 35]. These findings highlight the potential of Vc-enriched potatoes as a substantial dietary Vc source, even after common household cooking.

Comprehensive off-target analysis via whole-genome and shotgun sequencing confirmed the high specificity of the editing strategy. No off-target mutations were detected, although a low frequency of spontaneous nucleotide substitutions was observed (Fig. S6). To further rule out the possibility that the severe growth penalty in the *uorf-StGGP1-*B line was a consequence of unintended, gene-disrupting integration of the CRISPR/Cas9 T-DNA vector—rather than the intended uORF editing per se—we performed a dedicated bioinformatic screen for vector backbone sequences. Using the complete sequence of our transformation vector (pkse402) as a reference, we re-analyzed the whole-genome shotgun sequencing data from the wild-type and all key edited lines. No reads uniquely mapping to the vector backbone were detected in any edited line, including *uorf-StGGP1-*B (Table S2). This result conclusively demonstrates the absence of random, large-scale T-DNA integrations that could disrupt fitness-related genes. Therefore, the observed growth defect can be robustly attributed to the disruption of the *uorf-StGGP1* cis-regulatory element and the consequent Vc hyperaccumulation, rather than to off-target technical artifacts from the transformation process. Together, these results demonstrate that precise uORF editing can enhance tuber nutritional quality without compromising genetic stability, agronomic performance, or starch properties. In summary, moderate Vc enrichment through uORF editing achieves meaningful nutritional enhancement without the yield or quality penalties often linked with conventional enrichment approaches [36], thereby defining an optimal enrichment window for the development of functional food ingredients. In contrast, the severe growth defects observed in the high-accumulator *uorf-StGGP1*-B line underscore the existence of a threshold beyond which trade-offs become inevitable, warranting further investigation into the underlying mechanisms.

### Excessive vitamin C accumulation incurs growth penalties by disrupting auxin homeostasis

The development of potato lines with a gradient of Vc content established a critical threshold for beneficial enrichment. Moderate Vc enhancement was agronomically benign, whereas the high-accumulator line *uorf-StGGP1*-B (24.12-fold Vc increase) exhibited severe developmental penalties. This contrast provided a powerful system to dissect the molecular trade-offs between nutrient accumulation and plant growth. Furthermore, the observed abnormal pollen morphology and male sterility in *uorf-StGGP1*-B (Fig. S7A), consistent with previous reports in tomato [18, 37], underscore a conserved detrimental effect of Vc hyper-accumulation on reproductive development.

**Figure 5:**
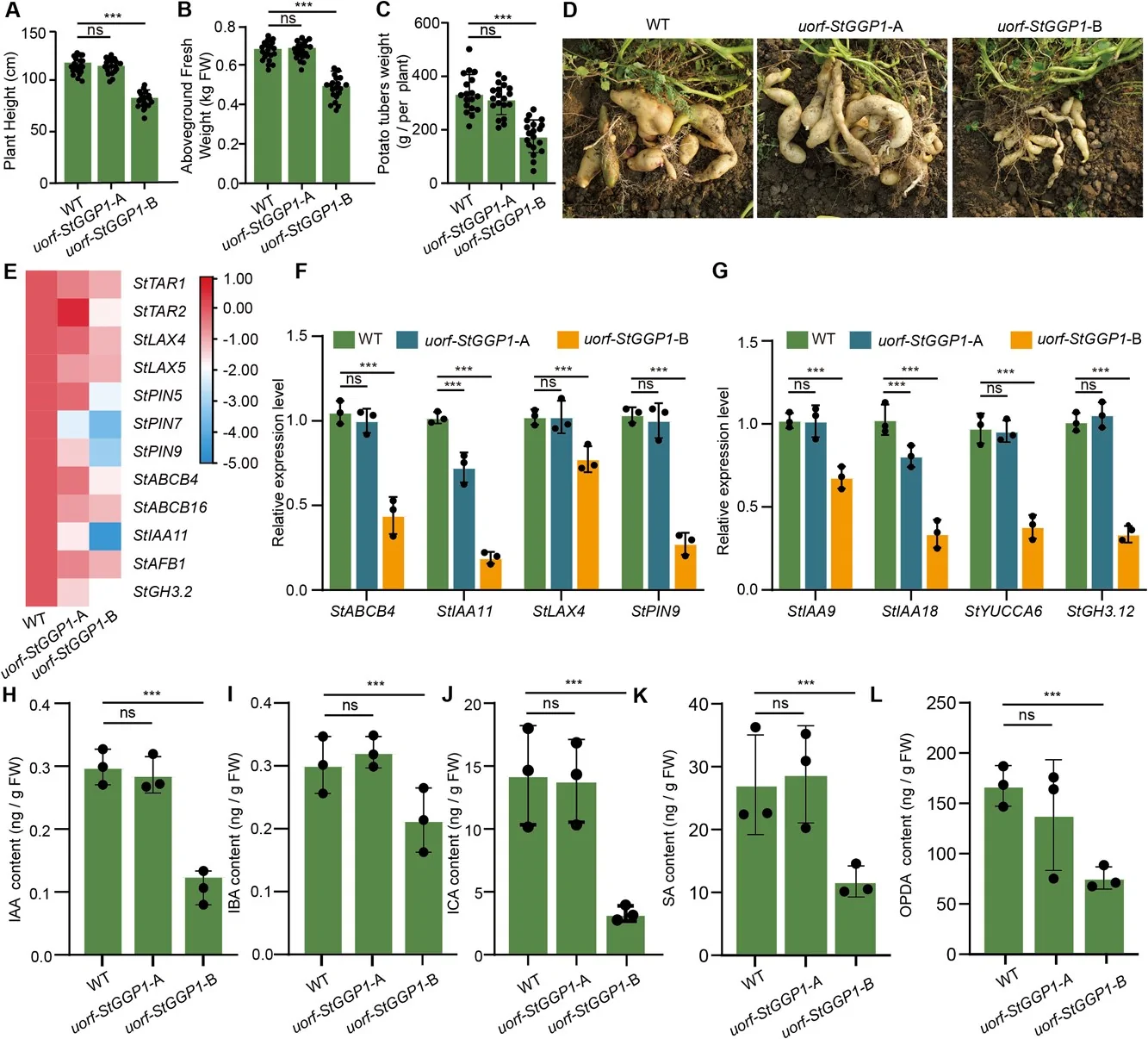
Excessive Vc accumulation leads to growth penalties. **(**A–C) Agronomic traits of WT, *uorf-StGGP1*-A and *uorf-StGGP1*-B lines from a greenhouse trial (spring 2022): (A) Plant height, (B) aboveground fresh weight, (C) tuber weight per plant. (D) Field trial phenotype (spring 2024, Dehong Field, Yunnan). (E) Heatmap of IAA synthesis/signaling genes in tubers. (F and G) qRT-PCR of auxin-related genes in tubers and leaves. (H–L) Endogenous phytohormone content in tubers: IAA (H), IBA (I), ICA (J), SA (K) and OPDA (L) content. All values: mean ± SD (tuber samples: *n* = 3 biological replicates). Data were analyzed by one-way ANOVA followed by Dunnett’s *post hoc* test. ^*^P <  0.05, ^**^P <  0.01, ^***^P <  0.001 vs WT control; ns, not significant.

More importantly, we observed rarely reported abnormalities in plant morphology and tuber development. From tissue culture onward, the *uorf-StGGP1*-B mutant exhibited delayed development, poor rooting rates, and overall stunted growth (Fig. S7B). At maturity, both plant height and above-ground biomass were significantly reduced relative to the WT and the moderate line *uorf-StGGP1*-A (Fig. 5A and B). These morphological abnormalities directly translated into significant yield loss, as confirmed by greenhouse trials over two seasons and a field trial (Fig. 5C and D; Fig. S8A and B). Additionally, slower tuber germination with a reduced number of buds indicated that extreme Vc levels disrupt tuber dormancy and sprouting (Fig. S7C). In contrast, *uorf-StGGP1*-A maintained normal growth, highlighting that such trade-offs are special to excessive Vc accumulation.

To elucidate the underlying mechanisms of growth penalties, we performed transcriptome profiling. Principal component analysis clearly segregated the transcriptomes of *uorf-StGGP1*-B from those of WT and *uorf-StGGP1*-A, indicating extensive transcriptional reprogramming (Fig. S9A and B). A substantial number of differentially expressed genes (DEGs) were specific to *uorf-StGGP1*-B, with 1025 DEGs in tubers and 4058 DEGs in leaves compared to WT, a significantly greater change than observed in *uorf-StGGP1*-A (Fig. S9C and D). KEGG pathway analysis highlighted a significant enrichment of DEGs in the tryptophan metabolism pathway, a primary route for auxin biosynthesis [38],observed specifically in both leaves and tubers of *uorf-StGGP1*-B (Fig. S9E–H).

Given the pivotal role of auxin in growth regulation [39, 40], we focused on auxin-related pathways in the high-Vc accumulator *uorf-StGGP1*-B. RNA-seq analysis revealed broad alterations in the expression of key genes involved in auxin biosynthesis (e.g., *TAR, YUCCA*), transport (e.g., *PIN, LAX, ABCB*), and signaling (e.g., *AUX/IAA, ARF, GH3*) (Fig. 5E–G; Fig. S10A). These transcriptomic findings were further validated by qPCR (Fig. 5F and G), which confirmed the significant downregulation of most auxin-related genes in *uorf-StGGP1*-B, consistent with the RNA-seq results.

This transcriptional disruption was corroborated by hormonal quantification. Compared with the wild-type (WT) and *uorf-StGGP1*-A lines, *uorf-StGGP1*-B tubers showed marked reductions in endogenous levels of indole-3-acetic acid (IAA; 0.46-fold), indole-3-butyric acid (IBA; 0.66-fold), and indole-3-carboxylic acid (ICA; 0.22-fold) (Fig. 5H–J). Similarly, IAA content was also reduced in the leaves of *uorf-StGGP1*-B (Fig. S10B and C). The suppression extended to salicylic acid (SA) and the jasmonate precursor 12-oxo-phytodienoic acid (OPDA), whereas other measured phytohormones were not significantly affected (Fig. 5K and L; Fig. S11). Collectively, these results indicate that Vc hyper-accumulation triggers a systemic phytohormone disruption.

**Figure 6:**
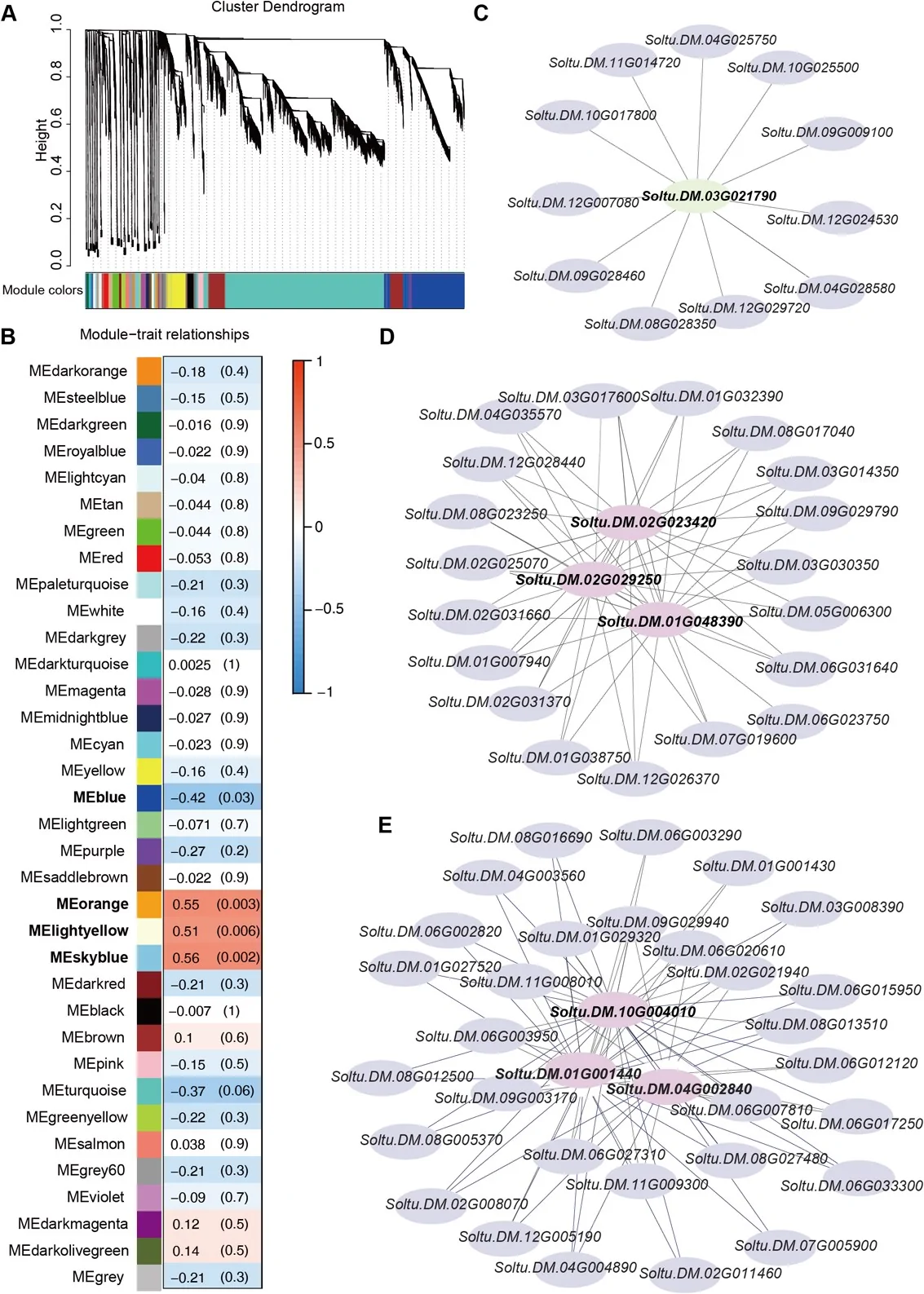
Module-trait relationships and gene co-expression network analysis. (A) Cluster dendrogram of co-expression modules identified by WGCNA. The WGCNA was constructed using transcriptomic data from both leaf and tuber tissues of the WT, *uorf-StGGP1-*A, and *uorf-StGGP1-*B lines. (B) Correlation analysis between gene co-expression modules and Vc content, showing correlation coefficients (color scale) and corresponding *P*-values (in parentheses). Key modules significantly associated with Vc accumulation are highlighted in bold. (C) Gene co-expression network of a representative negatively correlated module , with hub genes highlighted in bold. (D and E) Gene co-expression networks of representative positively correlated modules (D) and (E), with hub genes highlighted in large circles and bold.

The robustness of this inverse relationship was confirmed by weighted gene co-expression network analysis (WGCNA) (Fig. 6A). We identified a gene module (blue module) highly negatively correlated with Vc content and enriched for auxin-related genes (Fig. 6B and C). Conversely, modules positively correlated with Vc (e.g., sky-blue and orange module) were enriched for genes involved in ROS scavenging, such as peroxidases (Fig. 6B, D, and  E). This independent analysis underscores the fundamental trade-off: Vc hyper-accumulation is systemically linked to the suppression of auxin-mediated growth pathways, while being positively associated with antioxidant capacity.

In summary, our integrated physiological, transcriptomic, and hormonal data establish a compelling causal chain: excessive Vc accumulation triggers extensive transcriptional reprogramming that specifically disrupts the tryptophan-dependent auxin biosynthesis pathway and its associated signaling network. This systemic suppression of auxin, a master regulator of growth [41], contributes to the severe developmental penalties observed in the *uorf-StGGP1*-B line, defining a critical cost of nutrient hyper-accumulation.

### Exogenous auxin application partially rescues the growth defects of the high-Vc accumulator

To directly test whether the growth penalty in the *uorf-StGGP1-*B mutant was causally linked to auxin deficiency, we performed an exogenous auxin rescue assay. Seedlings of the wild-type (WT) and the *uorf-StGGP1-*B mutant, grown in tissue culture vessels, were treated with 10 μM IAA or a mock solution under controlled conditions.

Strikingly, exogenous IAA treatment led to a substantial but partial phenotypic recovery. Compared to the mock-treated *uorf-StGGP1-*B plants, which exhibited severe stunting, purple pigmentation (a sign of stress), and thin stems, the IAA-treated mutants showed a significant increase in plant height, stem diameter, and above-ground fresh weight, and displayed a greener, healthier appearance, although their overall size and vigor remained below that of the wild type (Fig. S12A–E). This marked improvement confirmed the causal role of auxin deficiency, while the incomplete rescue indicated that the growth penalty could not be fully reversed by external auxin supply.

We next examined the molecular response to this treatment by quantifying the expression of canonical auxin-responsive genes. qRT-PCR analysis revealed that the expression of *StIAA18*, which was downregulated in the mock-treated mutant, was restored to near-WT levels upon IAA application (Fig. S12F). In contrast, the expression of *StGH3.12* remained significantly suppressed even after rescue (Fig. S12G), suggesting a complex and persistent alteration in specific branches of the auxin signaling network within the *uorf-StGGP1-*B background.

Together, these results provide direct functional evidence that the severe growth penalty associated with Vc hyperaccumulation is caused, at least in part, by a deficiency in auxin response. The partial but significant phenotypic rescue achieved by external auxin supply confirms the central role of auxin disruption in this trade-off, while the incomplete molecular and phenotypic recovery suggests either auxin-independent effects or a deep-rooted signaling lesion that cannot be fully overcome by short-term hormone supplementation.

### Multifaceted benefits of Vc enrichment: enhanced antioxidant capacity and stress resilience

**Figure 7:**
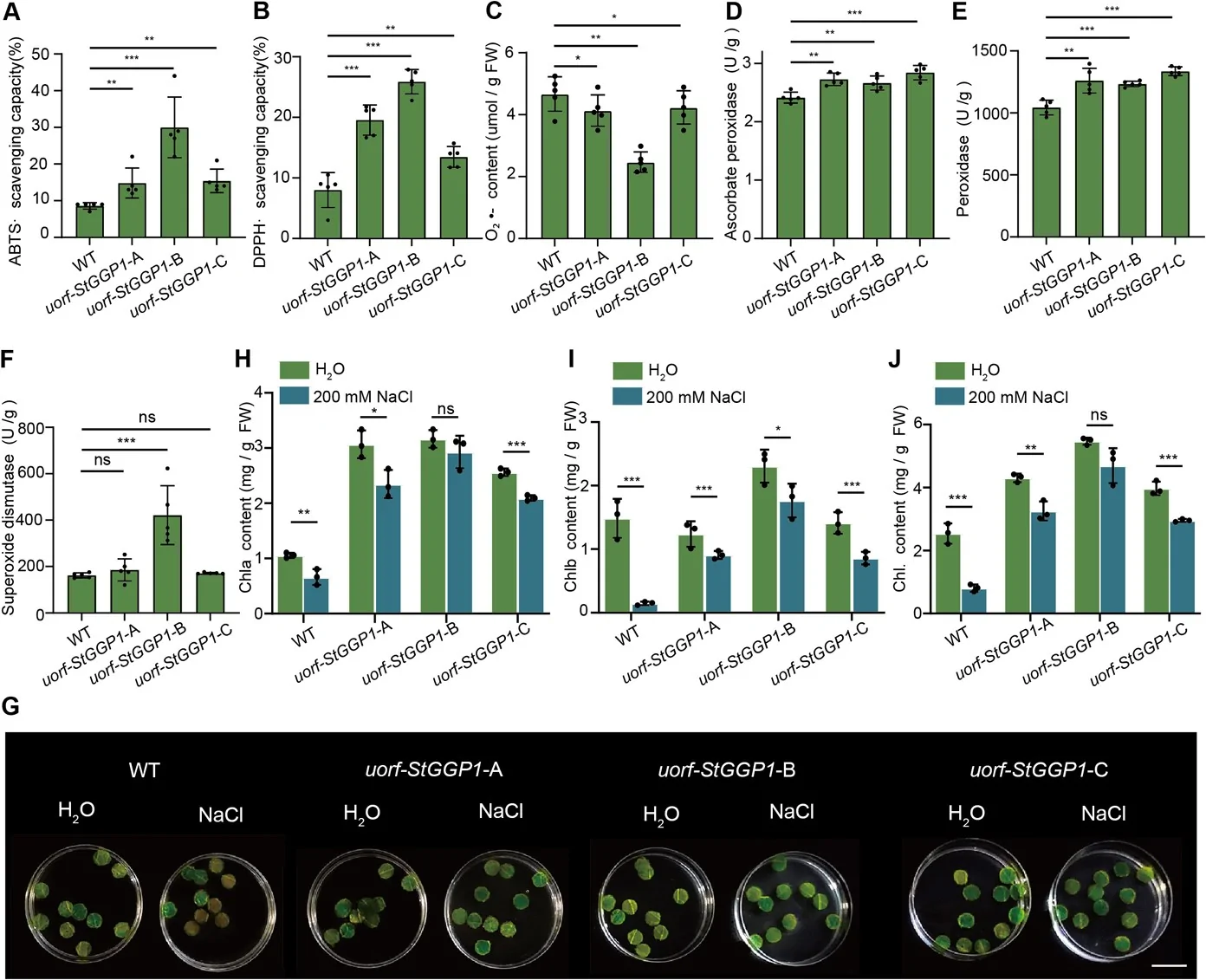
Antioxidant capacities and salt tolerance of WT and *uorf-StGGP1* edited lines. **(**A–F) Antioxidant indices in tuber samples: (A) ABTS radical scavenging activity, (B) DPPH radical scavenging activity, (C) O₂·^−^ content, (D) ascorbate peroxidase, (E) peroxidase, (F)superoxide dismutase. (G) Phenotype of leaf discs from WT and edited lines under 200 mM salt stress (4 days, continuous light). Scale bar = 15 mm. (H–J) Chlorophyll content in salt-treated leaf discs (4 days): (H) chlorophyll a, (I) chlorophyll b, (J) total chlorophyll. All values: mean ± SD (tuber: *n* = 5; leaf discs: *n* = 3 biological replicates). Data were analyzed by one-way ANOVA followed by Dunnett’s *post hoc* test. ^*^P <  0.05, ^**^P <  0.01, ^***^P <  0.001 vs wild-type (WT) control; ns, not significant.

We investigated whether Vc enrichment confers benefits beyond nutrition enhancement, focusing on antioxidant capacity, stress resilience, and postharvest stability. Tubers from edited lines displayed a significant, dose-dependent enhancement in antioxidant activity, as evidenced by elevated 2,2′-azino-bis(3-ethylbenzothiazoline-6-sulfonic acid) (ABTS) and 2,2-diphenyl-1-picrylhydrazyl (DPPH) radical scavenging rates, directly correlating with Vc accumulation (Fig. S13; Fig. 7A and B). Among the Vc-enriched lines, *uorf-StGGP1*-B exhibited the highest antioxidant activity, followed by *uorf-StGGP1*-A and *uorf-StGGP1*-C, consistent with their respective Vc concentrations. This robust antioxidant phenotype was further supported by reduced superoxide anion levels in *uorf-StGGP1*-B tubers (Fig. 7C), along with significantly increased activities of key antioxidant enzymes—ascorbate peroxidase (APX, Fig. 7D) and peroxidase (POD, Fig. 7E)—across all fortified lines compared to the WT. Superoxide dismutase (SOD) activity was also highest in *uorf-StGGP1*-B (Fig. 7F), indicating a reinforced enzymatic antioxidant system.

However, an interesting divergence was observed in leaf oxidative status. 3,3′-Diaminobenzidine (DAB) and nitroblue tetrazolium (NBT) staining revealed markedly deeper staining in *uorf-StGGP1*-B leaves compared to WT and *uorf-StGGP1*-A, suggesting elevated hydrogen peroxide and superoxide anion accumulation and implying heightened oxidative stress in leaves despite enhanced tuber antioxidant capacity (Fig. S10). Under salt stress (200 mM NaCl, 4 days), leaf discs of *uorf-StGGP1*-edited plants exhibited a pronounced stay-green phenotype with minimal chlorophyll degradation, whereas the WT discs showed visible yellowing (Fig. 7G). Quantification confirmed that total chlorophyll retention was significantly higher in the edited lines after stress (Fig. 7H–J), indicating that Vc enrichment helps preserve preserve photosynthetic function under stress [42, 43].

In summary, uORF-mediated Vc enrichment in potato delivers significant physiological benefits: it strengthens the intrinsic antioxidant properties of tubers and enhances the whole-plant tolerance to abiotic stress. These functional improvements highlight the potential of nutritionally enriched potatoes to maintain quality under adverse conditions.

## Discussion

### uORF editing: a precision tool for metabolic engineering in potato

**Figure 8:**
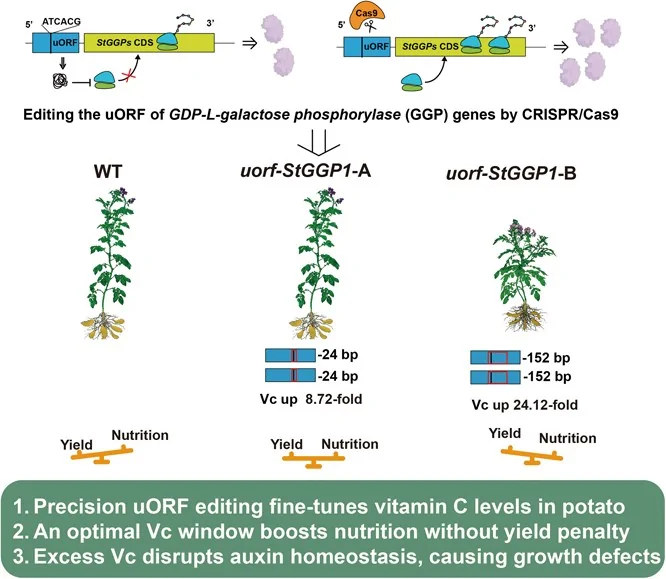
A schematic diagram illustrating the changes in Vc content mediated by CRISPR/Cas9-based genome editing of the *uorf-StGGP1*-A/B.

Our study establishes CRISPR/Cas9-mediated editing of uORFs as a precise and powerful strategy for Vc enrichment in potato (Fig. 8). By directly targeting translational repressors within the 5′ untranslated regions (UTRs) of the endogenous *GGP* genes, which encode the key rate-limiting enzyme in ascorbate biosynthesis [17], we achieved tunable enhancement of Vc accumulation. The efficacy of this approach in potato, consistent with foundational work in *Arabidopsis* [22], lettuce [21], and rice [44], validates uORF editing as a robust and generalizable platform for crop improvement.

A key advantage of this strategy is its remarkable tunability. We generated a spectrum of edited lines exhibiting Vc increases ranging from moderate (3.79- to 8.72-fold) to substantial (up to 24.12-fold), in contrast to the often extreme outcomes of constitutive overexpression [45–48]. The strong positive correlation between the size of the uORF deletion and the level of Vc enhancement underscores the dose-dependent and predictable nature of this methodology. This tunability represents a significant advance over constitutive overexpression, which frequently leads to metabolic imbalance and pleiotropic effects [49–51]. The strategy effectively transforms metabolic engineering from a simple on/off switch into a calibratable dial, enabling fine-tuning of endogenous gene expression without introducing foreign transgenes.

### A spectrum of phenotypes: agronomic excellence vs metabolic trade-offs

A central finding of our work is the delineation of an optimal enrichment window for Vc. Crucially, lines with moderate Vc enhancement (e.g., *uorf-StGGP1*-A), achieved through precise uORF editing, exhibited no compromise in key agronomic traits—including tuber yield, starch content and quality, and reproductive development. This demonstrates that substantial nutritional gains can be achieved without the yield penalties that often undermine conventional enrichment approaches [52].

In contrast, excessive Vc accumulation (e.g., 24.12-fold in *uorf-StGGP1*-B) led to observable metabolic imbalances and growth defects, illustrating a direct trade-off between high nutrient accumulation and agronomic performance [2, 53, 54]. The phenotypic spectrum we observed—from optimal performance to significant penalties—directly correlates with the level of Vc enhancement and underscores the critical importance of precise calibration.

### Dual benefits: enhanced nutritional quality and stress resilience

Beyond mere accumulation, our Vc-enriched potato lines exhibited significant functional advantages. The tubers displayed enhanced antioxidant capacity, a direct nutritional benefit. Furthermore, under oxidative stress, edited lines showed significantly reduced levels of ROS and malondialdehyde (MDA), alongside elevated activity of antioxidant enzymes such as superoxide dismutase (SOD) and ascorbate peroxidase (APX). This dual benefit of enrichment and stress tolerance mirrors findings in other species where ascorbate pathways were modulated [44, 55–57].

An essential consideration for Vc enrichment is nutrient stability during cooking. We found that the elevated Vc content demonstrated remarkable stability after steaming. We attribute this stability not to an alteration in Vc’s intrinsic thermal lability [58] but to a mass-action effect: the higher initial concentration ensures that a nutritionally substantial absolute amount remains postcooking, even after proportional degradation. This finding effectively transforms a perceived biochemical limitation into a practical nutritional solution.

### Vitamin C and auxin: a bidirectional antagonism governed by a critical threshold

Our study redefines the relationship between Vc and auxin. While it is known that auxin signaling can repress Vc biosynthesis to prioritize growth [5, 28, 29, 59], we demonstrate a bidirectional antagonism governed by a critical threshold. We show that excessive Vc accumulation in potato triggers a feedback loop that systemically disrupts auxin homeostasis. Exceeding a physiological Vc threshold transforms the known growth-directed suppression of antioxidants into a detrimental cycle that penalizes development.

Specifically, hyper-accumulation of Vc led to broad transcriptional reprogramming of the auxin pathway, impacting biosynthesis (e.g., *YUCCA*, *TAR*), transport (e.g., *PIN*, *ABCB*), and signaling components (e.g., *AUX/IAA*, *ARF*) [59]. This systemic compromise of auxin action provides a mechanistic basis for the severe growth defects in high-accumulator lines, reframing the classic growth–defense trade-off [37, 60] as not merely a passive metabolic burden but an active hormonal reprogramming initiated by excessive Vc, potentially mediated by redox signaling. This concept of a bidirectional antagonism, wherein excessive Vc triggers a feedback loop that suppresses auxin action, is strongly supported by independent evidence in *Arabidopsis*. A recent study demonstrated a transcriptional correlation between ascorbate levels and auxin biosynthesis genes, further substantiating the existence of a conserved Vc-auxin interplay [61]. Our work in potato critically extends this model by defining the physiological consequences of exceeding a critical threshold of this interplay in a major crop, establishing the ‘high-nutrition cost’ phenotype. Furthermore, the observed suppression of SA [62] and jasmonic acid precursors [63] suggests the hormonal impact extends beyond auxin, potentially compromising defense signaling.

We propose a model where Vc and auxin exist in a dynamic equilibrium. A critical Vc threshold acts as a switch: below it, auxin-dominated regulation supports growth; above it, a feedback-driven collapse of the auxin network occurs. This concept of a bidirectional antagonism with a critical threshold provides a fundamental physiological framework for designing enrichment strategies that avoid pleiotropic penalties by operating within safe metabolic boundaries. While our transcriptomic data strongly suggest a direct inhibitory role of supra-optimal Vc, the precise molecular mechanism—whether mediated through redox modification of auxin signaling components or alterations in auxin transport—remains a critical question for future research.

## Conclusion

This study establishes CRISPR-mediated uORF editing of *StGGP* genes as a precise and tunable strategy for Vc enrichment in potato. By generating a series of edited variants, we identified an optimal enrichment window—moderate Vc enrichment (3.79- to 8.72-fold)—that substantially increases tuber Vc content and remains nutritionally substantial after cooking, without compromising agronomic performance. Beyond this range, however, excessive Vc accumulation (>24.12-fold) leads to growth impairment, indicating that the conventional growth–defense trade-off is not inevitable but arises only when physiological limits are exceeded. Mechanistically, excessive Vc disrupts auxin biosynthesis and signaling, uncovering a bidirectional antagonism between Vc and auxin that is governed by a critical enrichment threshold. Our findings advance crop enrichment from proof-of-concept toward a strategic design process, validating uORF editing as a precise tool, and providing a physiological framework for enhancing nutritional traits in future crops without yield penalties.

## Materials and methods

### Plant materials

The potato variety S15–65 was obtained from the International Potato Center (CIP), while the *Désirée* cultivar was provided by Yunnan Normal University. Pot experiments were conducted with all WT and mutant plants grown in pots under controlled greenhouse conditions at the Institute for Vegetables and Flowers (IVF), Chinese Academy of Agricultural Sciences (CAAS), featuring a 16 h light/8 h dark photoperiod and diurnal temperatures of 25°C (day) and 18°C (night). Field experiments were performed in the experimental fields of the Dehong Prefecture Academy of Agricultural Sciences, Yunnan Province, China. For greenhouse-grown plants, tissues from three to five individuals of the same genotype were pooled to form a composite biological replicate for biochemical analyses; for field trials, data were collected from individual plants (*n* ≥ 10 per genotype) to account for environmental variability in phenotypic assessments.

### Plasmid construction and transformation

To confirm the accuracy of the uORFs in *Désirée* (a tetraploid cultivar) and S15–65 (a diploid line from the *Solanum tuberosum* group Phureja), experiments were conducted to amplify the *StGGP1*-uORF and *StGGP2*-uORF regions using the primers listed in Table S3. A 19-nt single guide RNA sequence for *StGGP1 uORF* and four 19-nt single guide RNA sequences for *StGGP2 uORF* were designed using the CRISPR-P tool (http://cbi.hzau.edu.cn/crispr) and constructed into pKSE402-AtU626. Using BsaI-digested pKSE402 as a backbone, sgRNAs were inserted into the vector. These sgRNAs were prepared using the primers listed in Table S3, which were designed to target specific genomic loci for gene editing. The final binary vectors, now containing the sgRNAs, were then used to transform potato internodes via *Agrobacterium tumefaciens*-mediated transformation using the EHA105 strain, as previously described [64]. To identify successfully transformed plants, GFP (green fluorescent protein) fluorescence was used as a marker, allowing for easy screening and selection of transgenic potato plants.

### Genotyping and ploidy analysis of transgenic plants

Leaf samples were collected from GFP (Green Fluorescent Protein) fluorescence-positive potato plants, and genomic DNA was extracted using the cetyltrimethylammonium bromide (CTAB) method. The targeted sequences were amplified from all regenerated plantlets using specific primers and then sequenced to identify mutations in the target regions. Individual lines were genotyped through sequencing of cloned PCR products. Since chromosome doubling occurs at a very high frequency during potato callus regeneration, the ploidy of mutated plants was determined using a flow cytometry assay (BD Aria SORP). At least twenty clones per sample were sequenced from tetraploid plants. Nuclear DNA content was analyzed by flow cytometry to screen for unintended ploidy changes during tissue culture regeneration. In the resulting histograms, the x-axis represents relative nuclear DNA content (fluorescence intensity). For a diploid (2C) cell population, two characteristic peaks are observed: a major peak at a lower fluorescence intensity corresponding to cells in the G0/G1 phase (with 2C DNA content) and a smaller peak at approximately twice that intensity corresponding to cells in the G2/M phase (with 4C DNA content). A uniform shift of both peaks to approximately double the fluorescence intensity (e.g., G0/G1 at 4C, G2/M at 8C) indicates a tetraploid (4C) population. The ploidy of all regenerated lines was confirmed by comparing their peak profiles with those of known diploid (S15–65) and tetraploid (Désirée) wild-type controls.

### Dual-luciferase reporter assays

To assay the effect of uORF mutations on translation, dual-luciferase reporter constructs were generated. Briefly, the 5′ leader sequences of *StGGP1* and *StGGP2* (including wild-type or CRISPR-edited uORFs) were amplified from genomic DNA of the corresponding potato lines, cloned into a T-vector for sequencing, and sequence-verified mutant clones were selected. The verified fragments were then directionally cloned (BamHI/NotI) into the binary vector pGreenII0800-LUC-35S, placing the *Firefly* luciferase (LUC) gene under the control of the CaMV 35S promoter preceded by the respective leader sequence. The vector also carries a constitutively expressed *Renilla* luciferase (REN) gene for normalization.

A series of point mutations were introduced into the uORFs to progressively disrupt their function. For *StGGP1* (G1), mutants included changes to the Kozak context (G1-M1: −3 A → C; G1-M2: +4 G → T), the start codon (G1-M3: ACG → TCG), a combination of the −3 Kozak and start-codon mutations (G1-M4), and an early in-frame stop codon as a control for complete uORF ablation (G1-M5). For *StGGP2* (G2), mutants comprised G2-M1 (+4 G → T), G2-M2 (start codon ACG → GCG), the combined mutant G2-M3 (−3 A → C & ACG → TCG), and an early stop-codon control (G2-M4).

For transient expression assays, the constructs were introduced into *A. tumefaciens* strain GV3101. Bacterial suspensions (OD600 = 0.6) were infiltrated into leaves of 4-week-old *Nicotiana benthamiana* plants. After 12 h of dark incubation, plants were grown for 2 days under a 16-h light/8-h dark cycle. Leaf discs were then harvested, and LUC and REN activities were quantified using the Dual-Luciferase® Reporter Assay System (Promega). Relative downstream translation was expressed as the LUC/REN activity ratio.

### RNA preparation and quantitative real-time PCR

Total RNA was extracted using the Quick RNA Isolation Kit following the manufacturer’s protocol. qRT-PCR was performed on a 7500 Fast Real-Time PCR System (Applied Biosystems) with SYBR Premix (Takara) or Eastep qPCR Master Mix (Promega), following respective kits’ instructions. All reactions were conducted with three biological replicates using primers listed in Table S3.

### Protein extraction, western blotting, and quantitative analysis

Fresh samples were flash-frozen in liquid nitrogen and ground to powder. Proteins were extracted using an RIPA-like buffer (50 mM Tris–HCl pH 8.0, 150 mM NaCl, 1 mM EDTA, 2% SDS, 10% glycerol, 1% β-mercaptoethanol, 1× protease inhibitor cocktail) at 1 mL per 0.1 g tissue. Extracts were vortexed for 1 min and centrifuged (12 000 × *g*, 10 min, 4°C), and the supernatants were mixed with 5× SDS loading buffer (1:4 ratio) prior to boiling (95°C, 10 min).

Proteins (10 μL / lane) were separated by 12% SDS-PAGE gels (160 V, 80 min) and transferred to PVDF membranes (300 mA, 70 min). Membranes were blocked in 5% skim milk in PBST for 30 min at room temperature, followed by incubation at 4°C overnight. After blocking, membranes were probed with the following primary antibodies diluted in PBST containing 0.5% skim milk for 1 h at room temperature: anti-GGP antibody (monoclonal, PhytoAB, USA, Cat# PHY0233S), used at a 1:2000 dilution. This commercially available antibody was raised against a conserved peptide sequence of plant GGP and has been validated for use in multiple plant species, including *Solanum*. Anti-actin antibody (monoclonal, Abmart, China, Clone 26F7) was used at a 1:5000 dilution. This antibody is a widely used plant actin loading control.

After washing, membranes were incubated with HRP-conjugated secondary antibodies (goat anti-rabbit for GGP, CWBIO, China, Cat# CW0103S; goat anti-mouse for Actin, CWBIO, China, Cat# CW0102S) at a 1:5000 dilution for 30 min, followed by chemiluminescent detection.

Protein bands were analyzed using ImageJ software (available at https://imagej.net/software/imagej/). The integrated density of each band was measured, and GGP abundance was normalized to the corresponding actin signal. The relative GGP levels in *uorf-StGGP1* lines were normalized to the S15–65 wild type, and those in *uorf-StGGP2* lines were normalized to the Désirée wild type.

### Measurement of Vc content

Mature leaves, stems, and tubers were flash-frozen in liquid nitrogen and ground to a fine powder. Vc was extracted using 100-mL extraction buffer (containing 74.45 mg EDTA, 286.65 mg TCEP, and 5 mL 98% orthophosphoric acid in Milli-Q water). Samples were vortexed for 30 s, incubated for 30 min at 4°C, and then centrifuged (12 000 × g, 30 min, 4°C). Supernatants were filtered through 4-mm hydrophilic PTFE syringe filters.

Vc quantification was performed via LC–MS/MS using an Acquity CSH C18 column (Waters) with gradient elution (0.2 mL/min). Mobile phases: (A) 0.1% formic acid in water; (B) methanol with 0.1% formic acid. Gradient program: 5%–45% B (3 min), 45%–95% B (50 s), 95% B (2 min), 5% B (2 min re-equilibration). Column temperature: 35°C. Injection volume: 15 μl.

Detection was performed on an Agilent 6490 system in negative ESI mode (3500 V ion spray voltage, 35 psi curtain gas, 350°C collision gas temperature) using multiple reaction monitoring (MRM). Analytes were identified by mass transitions ; MRM parameters are provided in Table S4.

The LC–MS/MS method for Vc quantification was validated. It showed excellent linearity (*R*^2^ > 0.999) from 0.001 to 1.0 μg/mL, with a LOD of 0.15 ng/mL and a LOQ of 0.5 ng/mL. Precision was excellent, with relative standard deviations (RSDs, *n* = 3) of 1.17% (unspiked matrix), 1.24% (low spike, 0.01 mg/mL), and 1.35% (high spike, 0.5 mg/mL). Accuracy was confirmed by recovery experiments: recovery at 0.5 mg/mL was 96.4% with negligible matrix effect (96.8%), while at 0.01 mg/mL, recovery was 79.2% with a matrix effect of 82.6%, indicating mild ion suppression—a common phenomenon in complex plant matrices. Importantly, the Vc levels in our engineered lines fell within the high-concentration range where accuracy is excellent and matrix effects are minimal, ensuring reliable quantification.

### Antioxidant assays

To assess potato leaf phenotypes at maturity, mature leaves were stained with DAB, NBT, and trypan blue staining solutions, with each sample replicated three times for statistical reliability. For antioxidant capacity evaluation, the ABTS and DPPH assays were conducted to measure radical scavenging activity, while the superoxide anion radical scavenging capacity was quantified via a colorimetric method [65]. The activity of superoxide dismutase (SOD) was assayed using the NBT photoreduction method, peroxidase (POD) activity via the guaiacol method, and ascorbate peroxidase (APX) activity through the ultraviolet spectrophotometric method, as described by Ali *et al.* [66].

### Extraction of starch content, determination of α-amylase activity, and electron microscopy observation of starch

The process starts with cleaning fresh potatoes to remove impurities. Following that, the potatoes are crushed into smaller pieces. The next step involves juice-residue separation, where the starch-rich juice is separated from the fibrous residue. Subsequently, ethanol precipitation is used to concentrate and purify the starch by precipitating it out of the liquid phase. The precipitated starch is then dried to remove excess moisture, resulting in the final starch product.

Using a kit from Beijing Solarbio Science & Technology Co., Ltd, we have measured relevant indicators related to α-amylase activity in potatoes. The freeze-dried samples were attached to aluminum stubs using adhesive double-sided carbon tape and subsequently coated with a gold layer via ion sputtering under vacuum conditions. This preparation method facilitated their examination under a scanning electron microscope (SEM) for detailed microscopic analysis.

### Measurement of chlorophyll content

Leaf discs (each 1 cm^2^) were precisely excised from the leaves and promptly submerged in aseptic water (serving as the control) or 200 mM NaCl solutions for a duration of 4 days. To extract chlorophyll (Chl) from these leaf samples, 80% ice-cold acetone was utilized. The absorbances of chlorophyll a (Chl a) at 664 nm and chlorophyll b (Chl b) at 647 nm were precisely quantified using a UV/Vis spectrophotometer. The total Chl content was subsequently calculated by summing the respective absorbances of Chl a and Chl b, after correcting for the specific extinction coefficients for each pigment at the respective wavelengths.

### Bioinformatics analysis of RNA-seq

Total RNA was extracted from seedling leaves (S15–65, *uorf-StGGP1*-A, *uorf-StGGP1*-B) and mature tubers for transcriptomic analysis. Illumina NovaSeq-generated 150-bp paired-end raw reads underwent quality control (QC) and filtering to obtain clean reads, which were aligned to the potato reference genome (http://solanaceae.plantbiology.msu.edu/index.shtml) using HISAT2. FeatureCounts processed BAM files for read quantification, followed by DESeq2 analysis to identify differentially expressed genes (DEGs; |fold change| ≥ 2, *padj* < 0.05). PCA was performed using R’s Psych package. Functional enrichment analysis of DEGs employed topGO, ClusterProfiler (KEGG), and OmicShare Tools, with visualization by GOplot (GO terms) and ggplot2 (KEGG pathways).

### Endogenous hormone extraction and quantification in potato tissues

Potato tubers and leaves were homogenized in extraction solvent (1:1 v/v 1% formic acid water: chromatographic-grade methanol) containing precooled internal standards (−20°C). Samples underwent 10-min sonication and 4-h shaking (metal bath, 4°C) for thorough compound release. After centrifugation (12 000 rpm, 10 min, 4°C), supernatants were filtered (0.22 μm) and transferred to LC–MS/MS vials.

Hormones were analyzed via UPLC-MS/MS using an Acquity UPLC® CSH C18 column (1.7 μm, 2.1 × 150 mm, Waters) with gradient elution (0.25 mL/min): 90% A (0.05% formic acid +2 mM ammonium formate)/10% B (0.05% formic acid in methanol) for 2 min, 30% B (2–4 min), 95% B (4–19 min), then re-equilibrated to 10% B (19.10–22 min) at 40°C (2 μl injection). Detection employed an AB Sciex Triple Quad 6500+ in MRM mode with optimized ESI parameters (±4500 V, adjusted source temperature/gas flows) for positive/negative modes. Each compound was measured in triplicate (parameters detailed in Table S5).

### Integrated analysis of transcriptome datasets

The genes from mature potato leaf and tuber tissues, having similar patterns, were identified by trend analysis, using MeV (version 4.9) [67], with the k-means method with *P* <  0.05. Expression values were calculated by dividing their expression level at all samples with their maximum observed count. Pearson’s correlation algorithm was used to construct gene regulatory networks.

### Co-expression network analysis

WGCNA was employed for co-expression network analysis, encompassing module division. Subsequently, further correlation analysis was conducted on the selected hub genes and key metabolites implicated in Vc content. The Pearson correlation coefficient was calculated to assess the relationship, and the co-expression network was visualized using Cytoscape software.

### Off-target analysis based on whole-genome resequencing

Genomic DNA was extracted from fresh leaves of each T_0_ plant and the wild-type (WT) control using the CTAB method. A standard genomic Illumina 150-bp paired-end library was produced from the chromosomal DNA. The extracted DNA was sequenced using an Illumina NovaSeq sequencer to a depth of at least 10 G by Novogene Gene Technology (Beijing). Off-target sites were predicted using the online tool Cas-OFFinder [68] by comparing the sgRNA target sites to the reference genome (Table S6). The CRISPResso [69] algorithm enables accurate quantification and visualization of genomic modifications in mutant plants, allowing for the filtering of background variation.

### Bioinformatic screening for vector backbone integration

To exclude phenotypic effects from random vector backbone integration, whole-genome sequencing reads from wild-type and edited lines were aligned simultaneously to the potato reference genome (*S.* tuberosum DM1–3 v6.1) and the complete *pkse402* transformation vector using BWA-MEM. Reads that mapped uniquely to the vector backbone (outside the T-DNA region) with MAPQ ≥20 were counted.

### Statistical analysis

For quantitative analysis of luciferase/Renilla luciferase activity, relative transcription level, plant height, aboveground fresh weight, tuber weight, leaf Vc content, stem Vc content, and tuber Vc content, at least six individual plants per genotype, or three biological replicates for each experiment, were used for statistical analysis. All numerical values are presented as means ± SD. Data were analyzed by one-way ANOVA followed by Dunnett’s *post hoc* test. ^*^P <  0.05, ^**^P <  0.01, ^***^P <  0.001 vs WT control; ns, not significant.

## Supplementary Material

Web_Material_uhag191

## Data Availability

All data supporting the findings of this study are available in the article or in Supplementary Information files, or are available from the corresponding author, upon request. Sequence data are present in The Arabidopsis Information Resource (TAIR) or solgenomics databases, under the following accession numbers: *AtGGP1* (*VTC2*) (*At4g26850*), *AtGGP2* (*VTC5*) (At5g55120), *SlGGP1* (*Solyc06g073320*), *SlGGP2* (*Solyc02g091510*), *StGGP1* (*Soltu.DM.06G028680*), *StGGP2* (*Soltu.DM.02G026810*). The raw sequencing data have been deposited in the NCBI Sequence Read Archive (SRA) under the BioProject accession number PRJNA1288594 (https://www.ncbi.nlm.nih.gov/bioproject/1288594).
