# Supplementary material for: Balancing tuber vitamin C improvement with trade-off side effect by fine-tuning regulation of StGGPs
Source: Hortic Res. 2026 May 13;13(10):uhag191. doi: 10.1093/hr/uhag191 (PMC13616495; doi:10.1093/hr/uhag191)
Supplement: Supplementary file 1 [file web_material_uhag191.zip › supplementry figure-Revised Tracked S1-S3.docx]

**Figure S1.** Conservative uORF prediction and structural analysis of *StGGP1* and *StGGP2*.
(A) Phylogenetic analysis of uORF sequences from dicot and monocot *GGP* genes. Potato *GGP* genes are highlighted (red line). The tree was constructed using MEGA7 (NJ method, 1000 bootstrap replicates). (B) Multiple sequence alignment of uORF proteins. Conserved motifs distinguishing monocot vs. dicot *GGP uORFs* are marked (red box).

**Figure S2.** Flow cytometry analysis of WT and *uORF*-edited lines (*StGGP1/StGGP2*).
(A)WT (S15 - 65) analysis. (B – D) *uorf - StGGP1* edited lines and WT (S15 - 65).(E) WT (Désirée) analysis. (F – I) *uorf - StGGP2* edited lines and WT (Désirée).

**(A, E)** Wild-type controls (diploid S15-65 and tetraploid Désirée) establish the baseline profiles. For the diploid S15-65 (A), the G0/G1 peak is at ~50 (2C DNA content) and the G2/M peak is at ~100 (4C DNA content). For the tetraploid Désirée (E), the corresponding peaks are shifted to ~100 (4C) and ~200 (8C), respectively. **(B-D, F-I)** All CRISPR-Cas9 edited lines exhibit flow cytometry profiles that are superimposable onto their respective wild-type controls. Specifically, the edited lines derived from the diploid background S15-65 (B-D) show the characteristic diploid peak pattern, while those derived from the tetraploid cultivar Désirée (F-I) show the characteristic tetraploid peak pattern. This demonstrates that the genome editing and regeneration process did not induce unintended ploidy changes in any of the lines analyzed.”

**Figure S3.** Quantitative dose–response analysis of uORF translation initiation efficiency in *StGGP1* and *StGGP2*.

(A) Dose–response curve for *StGGP1* (G1) uORF mutants. Relative downstream translation (LUC/REN activity) is shown for wild-type (G1-WT) and a series of point mutants designed to weaken uORF function. Mutants target the Kozak context (G1-M1: -3 A→C; G1-M2: +4 G→T) and the start codon (G1-M3: ACG→TCG). The G1-M4 mutant combines the -3 Kozak and start codon mutations. G1-M5 introduces an early in-frame stop codon as a control for complete uORF ablation. Data show a stepwise increase in LUC/REN activity as uORF function is weakened, with G1-M2 (weakened +4 Kozak) having the strongest effect among single mutants. Error bars represent mean ± SD (n = 3 biological replicates). Statistical significance was determined by one-way ANOVA with Dunnett's post-hoc test (*P < 0.001 vs. G1-WT).

(B) Dose–response curve for *StGGP2* (G2) uORF mutants. Analysis follows the same experimental design as in (A). Mutants include G2-M1 (+4 G→T), G2-M2 (start codon ACG→GCG), and the combined mutant G2-M3 (-3 A→C & ACG→TCG). G2-M4 is the early stop codon control. For StGGP2, the combined mutation (G2-M3) results in the strongest reduction of uORF-mediated repression. Error bars and statistical analysis are as described in (A).
