# Supplementary material for: Balancing tuber vitamin C improvement with trade-off side effect by fine-tuning regulation of StGGPs
Source: Hortic Res. 2026 May 13;13(10):uhag191. doi: 10.1093/hr/uhag191 (PMC13616495; doi:10.1093/hr/uhag191)
Supplement: Supplementary file 1 [file web_material_uhag191.zip › supplementry figure-Revised Tracked S10-S13.docx]

**Figure S10.** Expression of auxin-related genes and corresponding hormone quantification in leaves. (A) Heatmap of IAA biosynthesis and signaling gene expression in leaf tissues of *uorf-StGGP1-A/B* compared to the wild-type (WT). (B) Leaf indole-3-butyric acid (IBA) content. (C) Leaf indole-3-acetic acid (IAA) content.

**Figure S11.** Endogenous levels of selected phytohormones in tubers. Content of cis-Zeatin (CZ), dihydrojasmonic acid (DHJA), indole-3-propionic acid (IPA), trans-Zeatin (TZ), and glycosylated salicylic acid (SAG) in *uorf-StGGP1*-A/B compared to WT.

**Figure S12.** Exogenous auxin partially rescues the growth defects and gene expression in the high-vitamin C (Vc) accumulator *uorf-StGGP1-B* mutant.

(A-E) Phenotypic and growth parameters of wild-type (WT) and *uorf-StGGP1-*A/B mutant plants under mock or 10 µM indole-3-acetic acid (IAA) treatment. (A) Plant height, (B) stem diameter, (C) underground fresh weight, (D) above-ground fresh weight, and (E) representative phenotypes. levels of auxin-responsive marker genes in leaves. (F) Expression of *StIAA18*and (G) expression of *StGH3.12* in WT and *uorf-StGGP1-*A/B under mock or IAA treatment. Expression levels were normalized to the housekeeping gene and are shown relative to mock-treated WT. Data were analyzed by one-way ANOVA followed by Dunnett’s post-hoc test. **P* < 0.05, ***P* < 0.01, ****P* < 0.001 vs. wild-type (WT) control; ns, not significant.

**Figure S13.** Results of leaf reactive oxygen species staining using DAB (top) and NBT (bottom) in *uorf-StGGP1*-A/B and the WT. Scale bar=1.5 cm.
