# Supplementary material for: Balancing tuber vitamin C improvement with trade-off side effect by fine-tuning regulation of StGGPs
Source: Hortic Res. 2026 May 13;13(10):uhag191. doi: 10.1093/hr/uhag191 (PMC13616495; doi:10.1093/hr/uhag191)
Supplement: Supplementary file 1 [file web_material_uhag191.zip › supplementry figure-Revised Tracked S4-S6.docx]

**Figure S4.** Transcriptional and protein-level changes of leaves in WT (S15-65)/*uorf-StGGP1* and WT (Désirée)/*uorf-StGGP2* edited lines.

(A) Transcript levels of *StGGP1* and *StGGP2* mutants.(B) Protein levels of *StGGP1* and *StGGP2* mutants. (C) Semi-quantitative analysis of *StGGP* protein abundance.

**Figure S5.** Tuber yield per plant for GGP1 and GGP2 lines under greenhouse conditions.

**Figure S6.** The evaluation of off-target effects by whole-genome sequencing (WGS). The mutation patterns were detected in the ten most likely potential off-target (OT) sites for each sgRNA in WT and mutants. The pie charts represent the rate of the mutated reads at the OT site in each line.
