# Supplementary material for: Balancing tuber vitamin C improvement with trade-off side effect by fine-tuning regulation of StGGPs
Source: Hortic Res. 2026 May 13;13(10):uhag191. doi: 10.1093/hr/uhag191 (PMC13616495; doi:10.1093/hr/uhag191)
Supplement: Supplementary file 1 [file web_material_uhag191.zip › supplementry figure-Revised Tracked S7-S9.docx]

**Figure S7.** Pollen viability, sprouting phenotype, and sprouting sites in potato tubers.
(A) Mature pollen viability staining (scale bar = 200 μm). Genotypes: A (*uorf-StGGP1*-A), B (*uorf-StGGP1*-B). (B) Sprouting phenotype during tuber germination (scale bar = 5 cm). (C) Sprouting sites in tubers (white arrows; scale bar = 4 cm).

**Figure S8.**Tuber weight per plant and tuber yield under different growing conditions.

(A) autumn 2022 greenhouse experiment, (B) spring 2024 field trial in Dehong (Yunnan).

**Figure S9.** Transcriptome profiles of WT, *uorf-StGGP1*-A, and *uorf-StGGP1*-B in tuber and leaf tissues.(A) Principal component analysis (PCA) of tuber transcriptomes.(B) PCA of leaf transcriptomes.(C) Venn diagram showing differentially expressed genes (DEGs) in tubers.(D) Venn diagram showing DEGs in leaves.(E, F) KEGG pathway enrichment analysis of DEGs in *uorf-StGGP1*-B versus WT for leaf (E) and tuber (F) tissues.(G, H) KEGG pathway enrichment analysis of DEGs in *uorf-StGGP1*-A versus WT for leaf (G) and tuber (H) tissues.
